# Supplementary material for: Venomix: a simple bioinformatic pipeline for identifying and characterizing toxin gene candidates from transcriptomic data
Source: PeerJ. 2018 Jul 31;6:e5361. doi: 10.7717/peerj.5361 (PMC6074769; doi:10.7717/peerj.5361)
Supplement: Supplemental Information 3 [file peerj-06-5361-s003.gz › FinalOutput_E-20/Thrombin-like_enzyme_ancrod_11/finaltree.pdf]

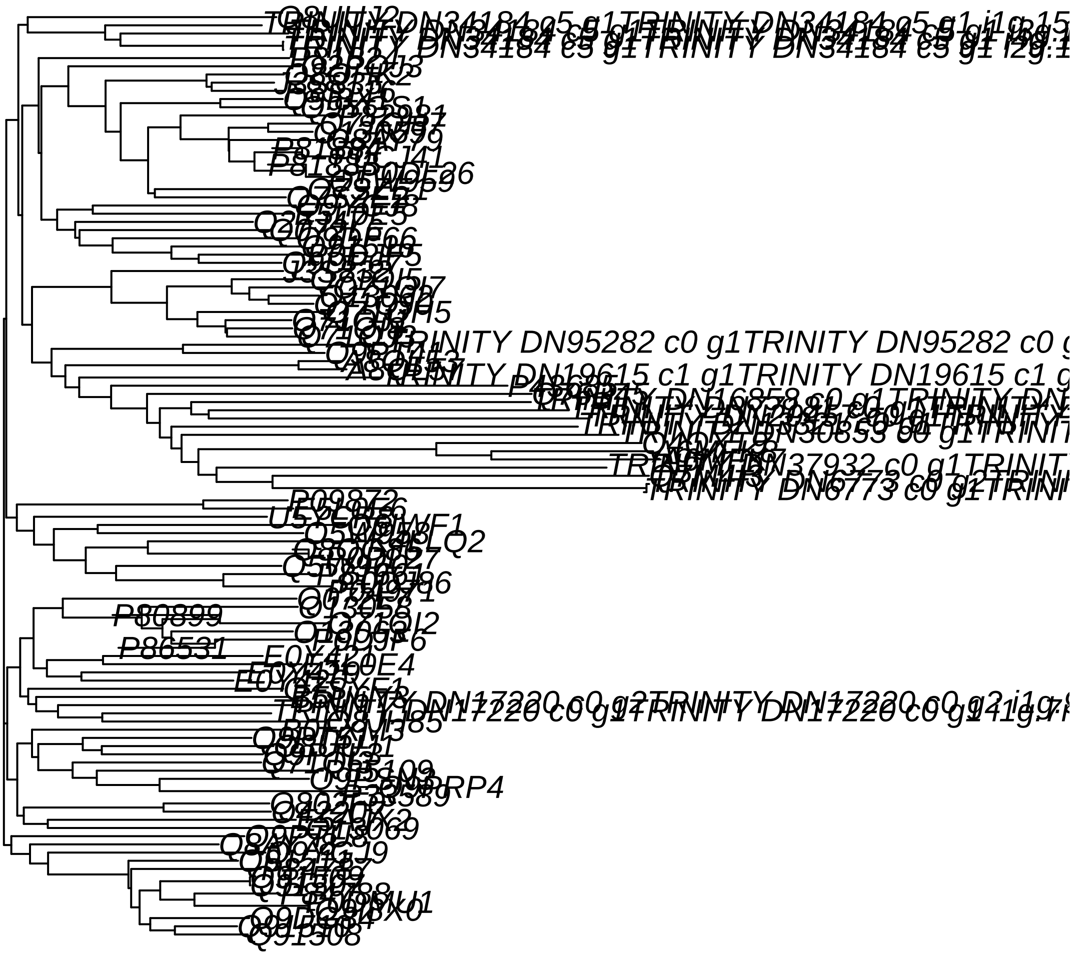
TRINITY DN95282 c0 g1TRINITY DN95282 c0 g
TRINITY DN19615 c1 g1TRINITY DN19615 c1 g
P09872
P80899
P86531
Q91508
